# Letter to “Levothyroxine absorption test results in patients with TSH elevation resistant to treatment”

**DOI:** 10.1007/s12020-021-02808-9

**Published:** 2021-07-20

**Authors:** Sadettin Öztürk, Ersin Akarsu

**Affiliations:** Department of Internal Medicine, Division of Endocrinology, Faculty of Medicine, Gaziantep University, Gaziantep, Turkey

We read the manuscript entitled “Levothyroxine absorption test results in patients with TSH elevation resistant to treatment” with great interest and pleasure [1]. We thought some points should be clarified so we decided to explain the test result discrepancies. İn the article to determine the absorbtion rate in patients with high levels of thyroid-stimulating hormone (TSH) despite receiving adequate doses of levothyroxine;

***% L4 absorbtion: [(peak ΔT4 × volume distribution) ÷ administered dose of LT(μg)] × 100***

***Volume distribution(dL): 4.42 × body mass index***

formula is used and according to the formula, those with an absorbtion result of more than 60–80% are considered normal. Considering the five patients evaluated in the study;

Absorption rates were not consistent with the data in the study Table 1. In addition, very high peakT4 values are required for the 60–80% absorption rate required for the test to be normal. In the study named “The clinical utility of free thyroxine in oral levothyroxine absorption testing”, in which the formula is taken as reference, calculations were made with total T4 and free T4 values and it was observed that similar incompatibility was detected in the same way [2].

**Table 1:** LT4 absorption test result

Patient	Basal fT4^a^	Peak fT4	BMI (kg/m^2^)	Absorption (%)
1	0.636	0.639	27	**6**
2	0.290	0.440	27	**40**
3	0.400	2.000	47	**166**
4	0.200	1.300	26	**97**
5	0.530	1.980	30	**90**

^a^Reference range of normal fT4 level is 0.89–1.76 ng/dL

Patient 1: [(0.639−0.636) × 4.42 × 27**÷**1000] × 100 = **%0.035**

Patient 2: [(0.440−0.290) × 4.42 × 27**÷**1000] × 100 = **%1.79**

Patient 3: [(2.000−0.400) × 4.42 × 47**÷**1000] × 100 = **%33.2**

Patient 4: [(1.300−0.200) × 4.42 × 26**÷**1000] × 100 = **%12.64**

Patient 5: [(1.980−0.530) × 4.42 × 30**÷**1000] × 100 = **%19.27**

Bold values in the table from orjinal study

As a result, the reliability of this formula, which is used to evaluate the absorption of levotroxin in patients with persistent elevation of TSH despite levothyroxine replacement therapy, is controversial and there are studies in which an increase of 50–100% in basal free T4 is accepted to evaluate malabsorption [3].
